# The current practice of handling and reporting missing outcome data in eight widely-used PROMS in RCT publications: are we doing well?

**DOI:** 10.1186/1745-6215-16-S2-P67

**Published:** 2015-11-16

**Authors:** Ines Rombach, Oliver Rivero-Arias, Crispin Jenkinson, Alastair Gray, Órlaith Burke

**Affiliations:** 1 Health Economics Research Centre (HERC), Nuffield Department of Population Health, University of Oxford, Oxford, UK; 2 RCS Surgical Intervention Trials Unit (SITU), Nuffield Department of Orthopaedics, Rheumatology and Musculoskeletal Sciences, University of Oxford, Oxford, UK; 3 National Perinatal Epidemiology Unit (NEPU), Nuffield Department of Population Health, University of Oxford, Oxford, UK; 4 Health Services Research Unit (HSRU), Nuffield Department of Population Health, University of Oxford, Oxford, UK; 5 Clinical Trial Service Unit and Epidemiological Studies Unit (CTSU), Nuffield Department of Population Health, University of Oxford, Oxford, UK

## Purpose

Patient reported outcome measures (PROMs) are designed to assess patients’ perceived health states or health-related quality of life. However, PROMs are susceptible to missing data, which can affect the validity of conclusions from randomised controlled trials (RCTs). This review aims to assess current practice in the handling, analysis and reporting of missing PROMs outcome data in RCTs in relation to contemporary methodology and guidance.

## Methods

This structured review of the literature includes RCTs with a minimum of 50 participants per arm. Studies using the EQ-5D-3L, EORTC QLQ-C30, SF-12, and SF-36 were included if published in 2013, those using the less commonly implemented HUI, OHS, OKS, and PDQ were included if published between 2009 and 2013.

## Results

The review included 209 papers (4 to 76 per relevant PROM). Complete case analysis and single imputation were commonly used in 33% and 15% of publications respectively. Multiple imputation was reported for 9% of the PROMs reviewed. The majority of publications (93%) failed to describe the assumed missing data mechanism, while low numbers of papers reported methods to minimise missing data (29%), performed sensitivity analyses (22%) or discussed the potential influence of missing data on results (16%).

## Conclusions

Considerable discrepancy exists between approved methodology and current practice in handling, analysis and reporting of missing PROMs outcome data in RCTs. Greater awareness is needed of the potential bias introduced by inappropriate handling of missing data, the importance of sensitivity analysis and clear reporting to enable appropriate assessments of treatment effects and conclusions from RCTs.

